# The ILEOSTIM trial: A multicentre randomised controlled trial evaluating the impact of efferent loop stimulation prior to ileostomy reversal on postoperative ileus

**DOI:** 10.1111/codi.70448

**Published:** 2026-05-06

**Authors:** Jorge Arredondo, Adriana Uriz, Irene Oliva, José I. Martín, Christian N. Iglesias, Ekta Choolani, Ainhoa Valle, Javier Rivera, Sebastián Jerí‐McFarlane, Juan M. Romero, Carolina González, Alicia Alvarellos, Marta Tasende, Lourdes Gómez, Laura Lázaro, Blanca Montcusí, Patricia Tejedor, Jeancarlos Trujillo‐Díaz, Alicia Ruiz de la Hermosa, Jorge Baixauli, Jorge M. Núñez‐Córdoba, Daniel Aliseda, ILEOSTIM Trial Group, Enrique Pastor, Jesús Fernández, Amaya Villafañe, María Beltrán, Ana Urioste, Herminia Lara, María Victoria Diago, Tania Gotor, Isabel Cifrian, Luis Miguel Jiménez, Elena Hurtado, Carlos Pastor, Fernando Labarga, Rosalía Velasco, Vicente Simó, Teresa Calderón, Mahur Esmailli, Patricia Ortega, María Luisa de Fuenmayor, Vanesa Serrano, Isabel Prieto, Lequerica Cabello, Carlos Sánchez, Alberto Bravo, Lucrecia Rodríguez, Juana Escudero, Alejandro Morales, Maria Cruz Correa, Naybet Pérez, Juan Carlos Martín, Pilar Concejo, Juan Ramón Gómez, Clara Martínez, Javier Atienza, Alejandro Gil, Margarita Gamundi, Aina Ochogavía, Enrique Colás, Naila Pagés, Susana González, Laia Cabré, Marta Pascual, Mayra Rebeka Abad, Isabel Seco, Víctor Valbuena, Rubén Caina, Estefanía Sánchez, Alicia Ferrer, Virginia Jiménez, Salvadora Delgado, Mireia Lázaro, Rubén Rodríguez, Adoración Meana, Paola Lora

**Affiliations:** ^1^ Department of General Surgery Clínica Universidad de Navarra Pamplona Spain; ^2^ Department of General Surgery Hospital Universitario de León León Spain; ^3^ Department of General Surgery Hospital Universitario Marqués de Valdecilla Santander Spain; ^4^ Department of General Surgery Hospital Universitario de Cabueñes Gijón Spain; ^5^ Department of General Surgery Hospital Universitario Río Hortega Valladolid Spain; ^6^ Department of General Surgery Hospital Universitario de Getafe Madrid Spain; ^7^ Department of General Surgery Hospital Universitario de Canarias Tenerife Spain; ^8^ Department of General Surgery Hospital Universitario Son Espases Palma de Mallorca Spain; ^9^ Department of General Surgery Hospital Universitari Mútua Terrasa Barcelona Spain; ^10^ Department of General Surgery Hospital Universitario La Paz Madrid Spain; ^11^ Department of General Surgery Clínica Universidad de Navarra Madrid Spain; ^12^ Department of General Surgery Hospital Universitario Son Llatzer Palma de Mallorca Spain; ^13^ Department of General Surgery Hospital Nuestra Señora del Prado Talavera de la Reina Spain; ^14^ Department of General Surgery Hospital Universitari Dexeus Barcelona Spain; ^15^ Department of General Surgery Hospital del Mar Barcelona Spain; ^16^ Department of General Surgery Hospital Universitario Gregorio Marañón Madrid Spain; ^17^ Department of General Surgery Hospital Medina del Campo Valladolid Spain; ^18^ Department of General Surgery Hospital Universitario Infanta Leonor Madrid Spain; ^19^ Research Support Service, Central Clinical Trials Unit Clínica Universidad de Navarra Pamplona Spain; ^20^ Division of General Surgery, Department of Surgery Stanford University Stanford California USA

**Keywords:** bowel stimulation, ileostomy reversal, loop ileostomy closure, postoperative ileus

## Abstract

**Background:**

Strategies to optimise bowel function before stoma reversal surgery (SRS) are needed to improve postoperative outcomes. Efferent bowel stimulation (EBS) prior to stoma closure has been proposed as a potential strategy to reduce postoperative ileus (POI) incidence and improve the patient's postoperative course.

**Methods:**

A multicentre randomised controlled trial was designed to evaluate the effectiveness of pre‐SRS EBS in reducing POI incidence. Patients scheduled for elective SRS were randomised 1:1 to either an intervention group, receiving daily EBS with saline and a nutritional thickener for 2 weeks preoperatively, or a control group, undergoing SRS without stimulation. The primary endpoint was POI incidence. Secondary outcomes included the time to oral diet tolerance, passage of first flatus/stool, surgery‐related comorbidities, length of hospital stay and incidence of Low Anterior Resection Syndrome (LARS) (NCT05302557‐31/03/2022).

**Results:**

From June 2021 to April 2023, 175 patients were enrolled from 18 hospitals in Spain, 78 (44.5%) and 97 (55.4%) of whom were allocated to the intervention and control groups, respectively. We found that EBS was not significantly associated with a decrease in POI incidence (7.6% vs. 16.4%, *p* = 0.088). No significant differences in the median hospital stay (*p* = 0.545) or LARS at discharge (*p* = 0.087) were observed between the two groups. The EBS group showed fewer but more severe complications (*p* = 0.015).

**Conclusion:**

Preoperative EBS is a feasible and reproducible intervention that may represent a potential strategy to reduce POI following SRS.

## INTRODUCTION

According to the most up‐to‐date epidemiological data from GLOBOCAN, rectal cancer (RC) is the eighth most prevalent form of cancer globally, with an estimated 729 833 new diagnoses annually. RC is the tenth‐leading cause of cancer‐related mortality (343 817 deaths in 2022) [1, 2]. The gold standard treatment for locally advanced RC is a combination of neoadjuvant chemoradiotherapy, surgery and adjuvant chemotherapy [3]. Advances in RC treatment, including the recent implementation of total neoadjuvant therapy (TNT) along with improvements in surgical techniques [4, 5], have allowed for more conservative sphincter‐preserving surgeries. These interventions, such as low anterior rectal resection with total mesorectal excision, prioritise the creation of anastomoses even in middle or distal rectal tumours.

This increase in the rate of lower (consequently complex) colorectal and coloanal anastomoses has resulted in a heightened risk of anastomotic leakage (AL) [6, 7, 8, 9]. In this high‐risk patient population, the estimated risk of AL, if no diversion stoma is performed, is 23% [10, 11, 12, 13, 14, 15], with an associated mortality rate of approximately 5% [9, 10, 12]. However, stoma creation has been shown to significantly reduce the risk of AL‐associated pelvic sepsis. Consequently, there has been a discernible increase in the number of diversion ileostomies performed in low anterior rectal resection procedures in recent years.

A diversion ileostomy, however, is not free of complications and has a negative impact on patients' quality of life [16]. Stoma reversal surgery (SRS) represents an additional surgical procedure and carries its own risk of morbidity. Previous studies have reported complication rates ranging from 18% to 37% following SRS [17, 18, 19], which may prolong hospitalisation and increase healthcare costs [20]. The most common complication after SRS is postoperative ileus (POI), with reported incidence rates between 12% and 28% [16, 17, 18, 19]. This complication is thought to result, at least in part, from histological and functional changes that occur in the excluded bowel during the diversion period [21]. One potential solution that has been posited is efferent bowel stimulation (EBS) before SRS. This proposed technique could serve as a method of diminishing dysbiosis, thereby enhancing intestinal absorption and contractility and consequently reducing POI incidence [22, 23, 24, 25].

Despite the extant evidence supporting the practice [22, 23, 24, 25], the implementation of EBS as a standard clinical practice remains contingent upon further investigation to ascertain its efficacy and determine the most appropriate stimulation protocol [22, 24, 25].

The aim of the ILEOSTIM trial was to evaluate whether EBS before SRS reduces the incidence of POI compared with standard reversal.

## MATERIALS AND METHODS

### Study design

An open‐label, multicentre, randomised controlled clinical trial was conducted to compare EBS (the intervention of interest) with direct surgery without stimulation (the control intervention). This study primarily aimed to analyse the impact of pre‐SRS EBS in terms of POI. The participants of this study were recruited by the principal investigators from 18 participating hospitals. The study was approved in April 2021 by the local Research Ethics Committee, with extensions to all participating centres (Ref: 2189). This study was conducted in accordance with the principles outlined in the Declaration of Helsinki to ensure the ethical protection and well‐being of its participants.

The study has been registered on ClinicalTrials.com (NCT05302557), and the trial protocol has been previously published elsewhere [26]. Participation in the study did not modify other surgical indications regarding the time to SRS, the type of intervention, or the anaesthesia employed, nor did it necessitate additional tests beyond those conducted in routine clinical practice. All patients were prospectively followed for at least 6 months after SRS. The results of this study were reported according to the CONSORT statement [27].

### Participants

Our inclusion criteria were as follows:

- Age >18 years.
- Candidacy for elective surgery to reverse the protective ileostomy created during RC surgery.
- Successful passage through a standardised protocol or rectal anastomosis evaluation, including a contrast enema, to exclude the presence of AL or stenosis according to standard clinical practice, before SRS.

The exclusion criteria for the study were as follows:

- Diversion ileostomy created for a pathology other than RC.
- A history of previous ileal surgery.
- Concomitant abdominal surgery (such as a cholecystectomy, hernia repair, or an appendectomy) at the time of SRS.

### Randomisation

Patients were randomised with a 1:1 ratio using the Sealed Envelope randomisation service©. The allocation sequence was concealed from the investigator enrolling study participants.

### EBS of the ileostomy

In the 2 weeks preceding SRS, patients in the intervention group underwent daily EBS through the irrigation of 500 mL of 0.9% saline solution combined with a nutritional thickener (Resource©, Health Science by Nestlé, 6.4 g sachet). A total of 10 sessions were scheduled, performed once daily until the eve of the planned surgery.

The first session was carried out under the supervision of the stoma nurse, who was responsible for instructing the patients on the correct technique. If the patient was self‐sufficient or had appropriate family support, the stimulation was subsequently performed at home. In cases where this was not feasible, patients were offered the option to return to the outpatient clinic to complete the sessions under supervision (Figure S1).

### Surgical technique and perioperative care

Stoma reversal entailed the creation of a skin elliptical incision around the stoma, meticulous dissection until full mobilisation of the stoma was attained, and the subsequent execution of the bowel anastomosis. Two anastomosis techniques were utilised according to the surgeon's preference:

1. Anti‐peristaltic side‐to‐side stapled anastomosis. In this case, the enterotomy should be closed with an additional stapled line and reinforced with a resorbable suture.
2. Hand‐sewn reversal with an end‐to‐end hand‐sewn anastomosis.

As this was a pragmatic trial, each surgeon implemented their routine surgical technique, choosing between options A and B in performing the anastomosis and using consistently the same technique for all their patients in the study, regardless of the group assigned during randomisation.

All patients received the routine protocol for prophylactic antibiotic therapy and anticoagulation as established in each centre. Routine postoperative antibiotic treatment was not required, following the Zero Surgical Infection protocol.

### Primary and secondary objectives

The primary objective of the study was to compare the post‐SRS POI incidence. POI was defined as intolerance to oral food intake on or after the third postoperative day, in the absence of clinical or radiological signs of obstruction, requiring nasogastric tube insertion or associated with at least two of the following: nausea, vomiting, abdominal distension and the absence of flatus [22, 24, 25].

The secondary objectives were as follows:

- Morbidity and mortality. They were defined as the occurrence of any deviation from the normal postoperative course during admission and the first 30 days after discharge, encompassing both symptomatic and asymptomatic events that negatively impact the patient's recovery. These were classified using the Clavien‐Dindo classification (CD) [28]. Major complications were described as CD ≥3. The readmission rate (the percentage of patients who had to be readmitted after being discharged during the first postoperative month) was also determined.
- Specific SRS outcomes:
  ○ Tolerance of a regular diet (the ability to digest solid and liquid foods without experiencing adverse effects).
  ○ First passage of stool and flatus (the postoperative day on which the patient passed gas or stool for the first time).
- Specific primary rectal surgery sequelae: Low Anterior Resection Syndrome (LARS) [29] was evaluated at discharge and at the first and sixth postoperative months.

### Statistical analysis

Assuming an alpha risk of 0.05 and a beta risk of 0.2, with a two‐tailed test, it was estimated that 68 patients in each group were needed to find a statistically significant difference in the proportions. A proportion of 0.29 was assumed in the control group [18, 19] and 0.1 in the intervention group [22, 24]. A 5% patient loss was anticipated.

Categorical variables were presented as frequencies with percentages and compared using contingency tables and the chi‐square test. Continuous variables were evaluated by comparing means using Student's *t*‐test and medians using the Mann–Whitney *U* test for normally and skewedly distributed variables, respectively. The threshold for statistical significance was set at *p* < 0.05. The statistical analyses were performed using Stata (StataCorp. 2023. Stata Statistical Software: Release 18. College Station, TX: StataCorp LLC).

Univariate and multivariate logistic regression analyses were performed to identify and quantify independent associations between the exposure variables and POI occurrence. Logistic regression analyses enabled the estimation of odds ratios with their corresponding 95% confidence intervals (CIs). The model was adjusted for covariates whose distributions differed significantly, both statistically and clinically, between the intervention and control groups and that could potentially influence POI occurrence.

## RESULTS

**FIGURE 1:**
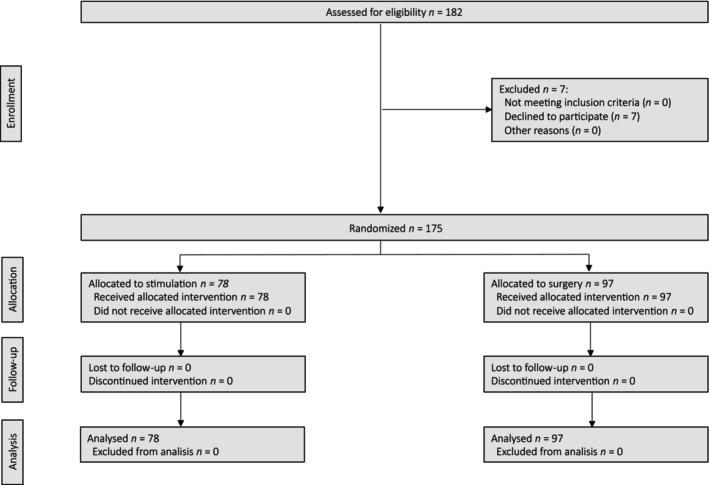
CONSORT diagram for the trial.

From 1 June 2021 to 15 April 2023, 175 patients were included in the study. EBS was performed in 78 (44.5%) of them before surgery, while the other 97 (55.4%) underwent SRS without prior stimulation (Figure 1).

### Baseline characteristics

The mean ages of the participants in the stimulation and control groups were 67 ± 10.9 and 68 ± 12 years, respectively. Sixty‐nine percent of the study participants were male (stimulation group: 73% vs. control group: 65%). The baseline characteristics of our study participants are detailed in Table 1.

**TABLE 1:** Baseline demographic and clinical characteristics of the patients.

	Stimulated group (*n* = 78)	Nonstimulated group (*n* = 97)	*p* *
Age (years)	67 (10.9)	68 (12)	0.844
Male sex (%)	57 (73.1)	64 (66)	0.312
BMI (kg/m^2^)	25.8 (4.8)	25.3 (3.8)	0.415
Analytical parameters			
Haemoglobin (g/dL)	13.7 (1.5)	13.4 (1.6)	0.242
Creatinine (mg/dL)	0.7 (0.5)	0.9 (0.3)	0.437
Total protein (g/dL)	6.8 (0.5)	6.8 (1.7)	0.350
Comorbilities			
Cardiovascular disease	24 (30.7)	21 (21.6)	
Renal disease	1 (1.2)	9 (9.2)	
Pulmonary disease	10 (12.8)	8 (8.2)	
Hypertension	32 (41)	44 (45.3)	
Diabetes mellitus	16 (20.5)	10 (10.3)	
ASA class (%)			0.817
1	4 (5.1)	7 (7.2)	
2	55 (70.5)	62 (63.9)	
3	18 (23)	26 (26.8)	
4	1 (1.2)	2 (2)	
Neoadyuvant therapy (%)	50 (64.1)	72 (74.2)	0.147
Time rectal surgery (min)	404.8 (281.1)	294.9 (221.2)	0.006
Postsurgical complications after rectal surgery	37 (47.4)	36 (37.1)	0.169
CD ≥ 3	15 (19.2)	5 (5.1)	0.004
Time from index rectal surgery to SRS (weeks)	57.8 (40.1)	41.7 (30.3)	0.004

*Note*: Data reported as *n* (%) or mean (SD), as appropriate.

Abbreviations: ASA, American Society of Anesthesiologists; BMI, body mass index; CD, Clavien‐Dindo classification.

* *p* < 0.05.

The distribution of three preoperative variables differed significantly between the two groups. Patients randomised to the EBS group had a longer operative time (404.8 vs. 294.9 min; *p* = 0.006), and a higher proportion of major complications (19.23% vs. 5.15%; *p* = 0.004) during primary tumour surgery, and a longer interval between RC surgery and SRS.

### Intraoperative characteristics

Regarding the type of anastomosis used for SRS, there was a higher percentage of mechanical anastomoses in the stimulation group than in the control group (87.17% vs. 64.94%, *p* = 0.001). The prevalence of parastomal hernia did not differ significantly between the two groups at the time of SRS (27 patients (34.6%) vs. 23 (23.7%), *p* = 0.121). The mean surgery duration did not differ significantly between the two groups (72.1 ± 25.6 min vs. 73.5 ± 30.3 min, *p* = 0.763).

### Postoperative outcomes

POI occurred in 6 patients (7.6%) in the EBS group and 16 patients (16.4%) in the control group (*p* = 0.088). An additional model adjusting for the type of anastomosis used during stoma reversal showed that EBS was associated with 53% lower odds of POI compared with no stimulation, although this association did not reach statistical significance (Odds ratio (OR): 0.47; 95% CI: 0.17–1.29; *p* = 0.142). Furthermore, when adjusted for the rate of major complications that occurred in the primary tumour surgery, the risk of POI was reduced by 62% in the EBS group (OR: 0.38; 95% CI: 0.13–1.07; *p* = 0.068) (Figure S2).

As secondary outcomes, the overall postoperative morbidity was 37.1% (65 patients). Although postoperative morbidity was slightly less frequent in the stimulated group (27 patients, 34.6%) compared to the nonstimulated group (38 patients, 39.2%), the stimulated group showed higher rates of major postoperative complications. These major complications included one episode of rectal bleeding, two anastomotic leaks, one abdominal wall bleeding, one evisceration, one bowel perforation and one episode of mesenteric bleeding requiring reoperation, which was complicated by multiorgan failure and death. In the control group, the major complication consisted of one anastomotic leak (Table 2).

**TABLE 2:** Postoperative morbidity.

	Stimulated group (*n* = 78)	Nonstimulated group (*n* = 97)	*p* *
Without postoperative morbidity	51 (65.4)	59 (60.8)	0.015
With postoperative morbidity CD^a^ < 3	20 (25.6)	37 (38.1)	
With postoperative morbidity CD^a^ ≥ 3	7 (9.0)	1 (1.0)	

*Note*: Data reported as *n* (%).

^a^ CD, Clavien‐Dindo Classification. Clavien‐Dindo classification for postoperative morbidity in stimulated group (*n* = 27, 34.6%): 1 (*n* = 17, 63.0%), 2 (*n* = 3, 11.1%), 3A (*n* = 1, 3.7%), 3B (*n* = 3, 11.1%), 4 (*n* = 2, 7.4%), 5 (*n* = 1, 3.7%). Clavien‐Dindo classification for postoperative morbidity in the nonstimulated group (*n* = 38, 39.2%): 1 (*n* = 21, 55.3%), 2 (*n* = 16, 42.1%), 3A (*n* = 0), 3B (*n* = 1, 2.6%), 4 (*n* = 0), 5 (*n* = 0).

* *p* < 0.05.

The incidence of postoperative colitis was lower in the stimulation group than in the control group (0/78 [0%] vs. 5/97 [5.15%]), although the difference did not reach statistical significance (*p* = 0.066).

During mid‐term follow‐up, two patients in the control group presented late dehiscence of the rectal anastomosis, requiring one new ileostomy and one Hartmann procedure, respectively.

With respect to specific SRS outcomes, the mean time to the first passage of flatus was 1.7 ± 1 days in the stimulation group versus 1.8 ± 1.2 days in the control group (*p* = 0.768), and the mean time to the initiation of regular diet tolerance was 2.7 ± 1.2 days in the stimulation group versus 3 ± 2.2 days in the control group (*p* = 0.178). The mean time to the first stool passage was 3.12 ± 1.4 days in the stimulation group versus 2.54 ± 1.1 days in the control group (*p* = 0.005; Table 3).

**TABLE 3:** Specific stoma reversal surgery outcomes.

	Stimulated group (*n* = 78)	Nonstimulated group (*n* = 97)	*p*
Time to tolerate regular diet, days	2.7 (1.2)	3 (2.2)	0.178
Time to first flatus, days	1.7 (1)	1.8 (1.2)	0.768
Time to passage of first stool, days	3.1 (1.4)	2.5 (1.1)	0.005

*Note*: Data reported as mean (SD).

The median hospital stay duration was 4 days (p25–75: 3.0–5.0) in the stimulation group and 4 days (p25–75: 3.0–6.0) in the control group. The readmission rate was lower in patients who underwent stimulation: 4 patients (5.1%) vs. 10 patients (10.3%) in the stimulation and control groups, respectively (*p* = 0.209).

**FIGURE 2:**
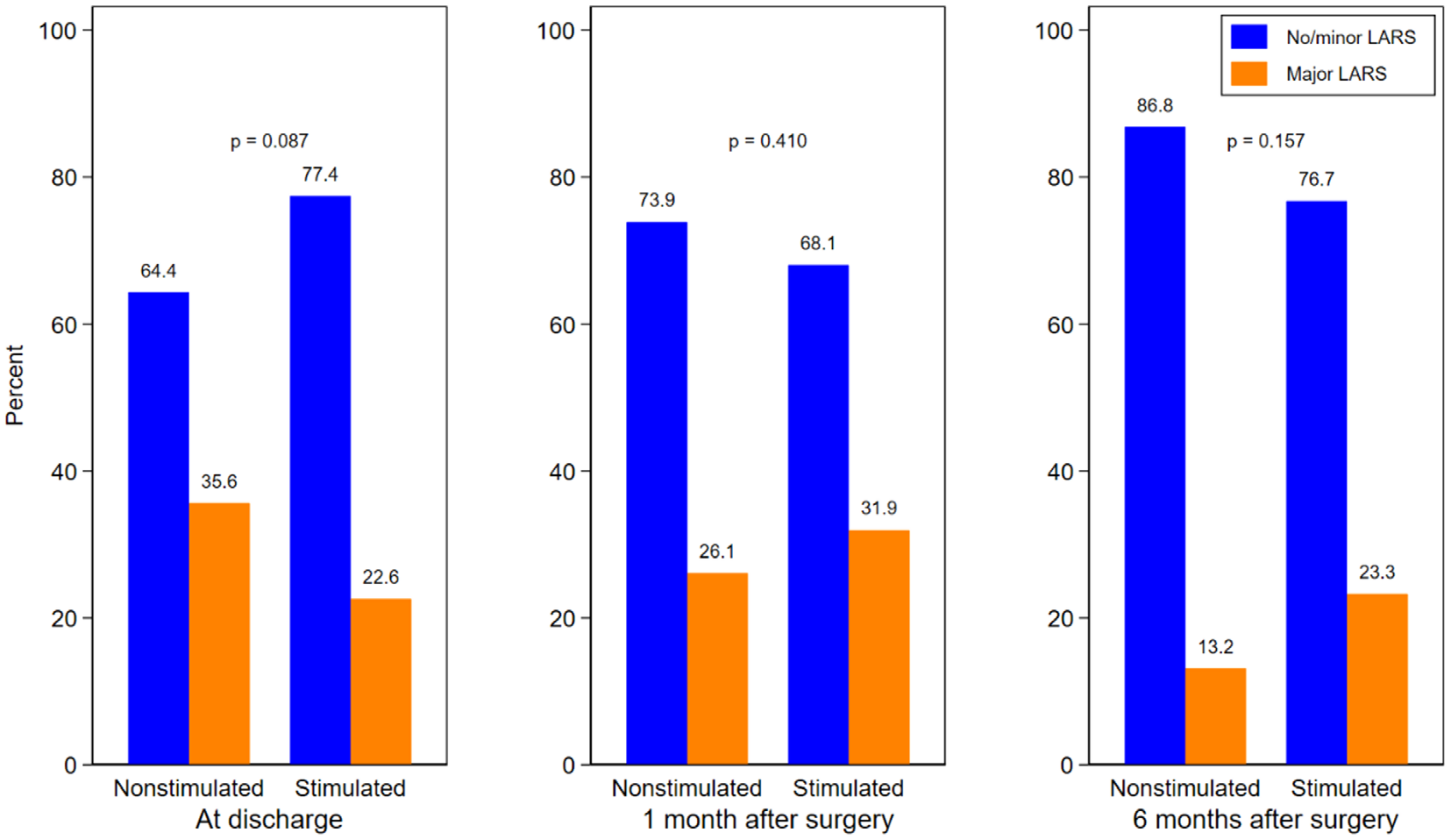
Low Anterior Resection Syndrome (LARS) score at discharge and at 1 and 6 months after surgery.

The entire sample showed a similar progression in LARS incidence at discharge (22.6% vs. 35.6%, *p* = 0.087), at one month (31.9% vs. 26.1%, *p* = 0.410) and at six months (23.3% vs. 13.2%, *p* = 0.157) (Figure 2).

## DISCUSSION

In this multicentre, open‐label, randomised controlled trial, EBS was associated with a 53.7% relative reduction in the rate of POI compared with standard care. Although this difference did not reach statistical significance, EBS appeared to be a safe and reproducible technique.

Several randomised clinical trials have described the stimulation procedure [22, 24, 25]. Two of these three studies demonstrated a significant reduction in POI occurrence in the stimulation group [22, 24]. Abrisqueta et al. conducted a randomised, single‐centre clinical trial with 70 patients (35 in the stimulation group), demonstrating a significant reduction in POI in the stimulated patients (2.9% vs. 20%, *p* = 0.024). Garfinkle et al. included 96 patients in their study, of which 47 underwent the stimulation protocol, also demonstrating a significant reduction in POI incidence in the stimulation group. Despite the positive results of these studies, one shared limitation of theirs is the small number of patients included. In contrast, in the ILEOSTIM trial, we successfully completed the estimated sample size of 175 patients across multiple centres. Although statistical significance was not reached, the observed >50% reduction in POI incidence in the stimulation group is clinically meaningful and consistent with the direction and magnitude of effect reported in previous studies. An additional limitation in the literature evaluating POI after SRS is the heterogeneity in POI definitions, with the incidence of POI being higher in those who had a specific definition [19]. To minimise this potential source of bias, POI was defined a priori using objective criteria, consistent with definitions applied in previous studies evaluating EBS [22].

To the best of our knowledge, there is currently no consensus on the most appropriate stimulation protocol, type of stimulant, or optimal duration of stimulation [4]. In our study, the patients in the stimulation group underwent daily EBS with 500 mL of saline solution combined with a nutritional thickener for the 2 weeks that preceded SRS, similar to the studies conducted by Abrisqueta et al. and Garfinkle [22, 24], with the latter implementing one additional week of stimulation. Other studies have used probiotics instead of saline with a thickener in the EBS protocol [23, 25], without obtaining a significant reduction in POI incidence in the stimulation group. This suggests that future studies comparing both stimulants would be interesting [4].

The creation of a diverting ileostomy leads to histological and functional changes contributing to POI development. Following SRS, the previously inactive colon must regain function and recover from intestinal atrophy while the entire gastrointestinal system readjusts, a process that may be associated with diarrhoea and colitis. EBS may modulate colonic inflammation. In our trial, we observed a lower incidence of this secondary clinical outcome in the stimulation group. Histological analysis and microbiota assessment could be of interest in the design of future studies aimed at better characterising the degree of colitis following EBS.

In the ILEOSTIM trial, no relevant EBS‐related complications were detected, as the integrity of the anastomosis was assessed prior to initiating stimulation, and tolerance to the procedure was high, likely facilitated by the close involvement of specialised stoma care nurses. These findings support the safety and feasibility of EBS in routine clinical practice.

We observed that patients randomised to the stimulation group had experienced more major complications after primary tumour surgery than those in the control group. From a theoretical standpoint, this may reflect a higher burden of adhesions and an increased baseline risk of POI in this group. Analysing the incidence of POI adjusted for complications secondary to primary tumour surgery, we observed that the risk of POI was reduced by 62% in the EBS group. In addition, although previous literature has suggested that handsewn versus stapled anastomoses may influence the risk of POI, adjustment for anastomosis type did not materially alter the effect estimate [20, 30, 31] These findings underscore the importance of accounting for surgical and perioperative factors when interpreting POI outcomes and support the need for stratified analyses by anastomosis type in future trials.

In 2014, Lloyd et al. conducted a systematic review and meta‐analysis including three randomised clinical trials, analysing the primary outcome of POI incidence after SRS [4]. This meta‐analysis did not reveal a significant reduction in POI incidence in patients who underwent EBS compared with those in the control group. However, reductions in time to oral intake, length of hospital stay and postoperative complications were reported. These findings were not fully replicated in our study, as although oral intake was initiated earlier in the stimulation group, the length of hospital stay was similar between groups and an earlier first bowel movement was observed in the control group. This discrepancy may be due to the lack of precision in secondary outcome measurement, as data were collected by postoperative day rather than by hours.

Although overall postoperative morbidity was slightly lower in the stimulation group, major complications were more frequent and more severe. After careful individual case review, these complications did not appear to be directly related to the efferent bowel stimulation procedure itself, nor to the stimulation protocol. Rather, they were attributable to factors inherent to the surgical procedure or the patients' baseline clinical characteristics, and therefore should be interpreted with caution when assessing the safety profile of the intervention.

With respect to functional outcomes, although no statistically significant differences were observed, the lower LARS rates in the stimulated group are hypothesis‐generating. It may be speculated that a longer duration of stimulation, beyond the two‐week protocol used in this study, might have a more pronounced impact on early postoperative bowel function, including LARS at discharge. At longer follow‐up, functional outcomes would be expected to converge between groups.

This trial was conducted using an intention‐to‐treat design to provide a pragmatic assessment of EBS effectiveness in a real‐world population, thereby avoiding overestimation of treatment effects. Several limitations should be acknowledged. First, adherence to the stimulation protocol was not formally assessed, and partial compliance may have contributed to an underestimation of the intervention's true effect; this should be carefully considered in the design of future protocols. Second, despite randomisation, a discrepancy in group sizes was observed, which might have been mitigated through block randomisation. Finally, longer operative times, greater severity of complications during primary tumour surgery and a longer interval between surgeries were observed in the stimulation group. These factors may have adversely influenced the effectiveness of EBS and should be taken into account when interpreting the study results.

## CONCLUSION

Preoperative EBS is a feasible and reproducible intervention that may represent a potential strategy to reduce POI following SRS.

## AUTHOR CONTRIBUTIONS

**Jorge Arredondo:** Conceptualization; data curation; investigation; methodology; project administration; resources; visualization; supervision; writing – original draft; writing – review and editing. **Adriana Uriz:** Conceptualization; investigation; methodology; supervision; visualization; writing – original draft; writing – review and editing. **Irene Oliva:** Conceptualization; data curation; investigation; methodology; project administration; writing – original draft; writing – review and editing. **José I. Martín:** Data curation; writing – review and editing. **Christian N. Iglesias:** Data curation; writing – review and editing. **Ekta Choolani:** Data curation; writing – review and editing. **Ainhoa Valle:** Data curation; writing – review and editing. **Javier Rivera:** Data curation; writing – review and editing. **Sebastián Jerí‐McFarlane:** Data curation; writing – review and editing. **Juan M. Romero:** Data curation; writing – review and editing. **Carolina González:** Data curation; writing – review and editing. **Alicia Alvarellos:** Data curation; writing – review and editing. **Marta Tasende:** Data curation; writing – review and editing. **Lourdes Gómez:** Data curation; writing – review and editing. **Laura Lázaro:** Data curation; writing – review and editing. **Blanca Montcusí:** Data curation; writing – review and editing. **Patricia Tejedor:** Data curation; conceptualization; methodology; writing – review and editing. **Jeancarlos Trujillo‐Díaz:** Data curation; writing – review and editing. **Alicia Ruiz de la Hermosa:** Data curation; writing – review and editing. **Jorge Baixauli:** Data curation; writing – review and editing. **Jorge M. Núñez‐Córdoba:** Investigation; methodology; writing – review and editing. **Daniel Aliseda:** Conceptualization; data curation; investigation; methodology; supervision; visualization; writing – review and editing; writing – original draft.

## FUNDING INFORMATION

No financial compensation was provided for participation in this study, either to the patients or the research team. The study was scientifically supported by the Spanish Association of Coloproctology (AECP) as a GECO‐4 project.

## CONFLICT OF INTEREST STATEMENT

The authors declare no conflicts of interest.

## ETHICS APPROVAL

This study was approved by the local research ethics committee (IRB). Patients could withdraw their consent at any time without negative consequences on the medical care they received thereafter. Moreover, the study was conducted in accordance with the principles outlined in the Declaration of Helsinki.

## INFORMED CONSENT

All participants gave their written informed consent before their enrolment in the study.

## CLINICAL TRIAL REGISTRATION

ClinicalTrials.gov Identifier: NCT05302557.

## AI STATEMENT

No generative AI tools were used in the writing or preparation of this manuscript.

## Supporting information


**Figure S1.** Ileostomy stimulation.
**Figure S2.** Crude and adjusted comparisons of primary outcome (postoperative ileus) by study arm. 95% CI, 95% confidence interval; OR, odds ratio. *Adjusted for postsurgical complications after rectal surgery (Clavien‐Dindo Classification ≥3). Reference: nonstimulated group.
**Data S1.** CONSORT checklist.

## Data Availability

The data generated and analysed during the present study are available from the corresponding author upon reasonable request.

## Appendix

Enrique Pastor; Jesús Fernández; Amaya Villafañe; María Beltrán; Ana Urioste; Herminia Lara; María Victoria Diago; Tania Gotor; Isabel Cifrian; Luis Miguel Jiménez; Elena Hurtado; Carlos Pastor; Fernando Labarga; Rosalía Velasco; Vicente Simó; Teresa Calderón; Mahur Esmailli; Patricia Ortega; María Luisa de Fuenmayor; Vanesa Serrano; Isabel Prieto; Lequerica Cabello; Carlos Sánchez; Alberto Bravo; Lucrecia Rodríguez; Juana Escudero; Alejandro Morales; Maria Cruz Correa; Naybet Pérez; Juan Carlos Martín; Pilar Concejo; Juan Ramón Gómez; Clara Martínez; Javier Atienza; Alejandro Gil; Margarita Gamundi; Aina Ochogavía; Enrique Colás; Naila Pagés; Susana González; Laia Cabré; Marta Pascual; Mayra Rebeka Abad; Isabel Seco; Víctor Valbuena; Rubén Caina; Estefanía Sánchez; Alicia Ferrer; Virginia Jiménez; Salvadora Delgado; Mireia Lázaro; Rubén Rodríguez; Adoración Meana; Paola Lora.
