# Supplementary material for: The ILEOSTIM trial: A multicentre randomised controlled trial evaluating the impact of efferent loop stimulation prior to ileostomy reversal on postoperative ileus
Source: Colorectal Dis. 2026 May 6;28:e70448. doi: 10.1111/codi.70448 (PMC13150047; doi:10.1111/codi.70448)
Supplement: Supplementary file 1 — Figure S1. Ileostomy stimulation. Figure S2. Crude and adjusted comparisons of primary outcome (postoperative ileus) by study arm. 95% CI, 95% confidence interval; OR, odds ratio. *Adjusted for postsurgical complications after rectal surgery (Clavien‐Dindo Classification ≥3). Reference: nonstimulated group. Data S1. CONSORT checklist. [file CODI-28-0-s001.zip › ILEOSTIM Trial Group.docx]

***ILEOSTIM Trial Group:*** **Hospital Universitario de León.** Enrique Pastor, Jesús Fernández,Amaya Villafañe, María Beltrán, Ana Urioste, Herminia Lara, María Victoria Diago, Tania Gotor, Isabel Cifrian. **Hospital Universitario Gregorio Marañón. Madrid**. Luis Miguel Jiménez y Elena Hurtado. **Clínica Universidad de Navarra. Madrid.** Carlos Pastor. **Hospital Universitario Río Hortega. Valladolid:** Fernando Labarga, Rosalía Velasco y Vicente Simó. **Hospital Nuestra Senora del Prado. Talavera de la Reina.** Teresa Calderón y Mahur Esmailli. **Hospital Universitario Infanta Leonor de Madrid.** Patricia Ortega, María Luisa de Fuenmayor y Vanesa Serrano. **Hospital Universitario La Paz. Madrid.** Isabel Prieto. **Hospital Universitari Dexeus. Barcelona.** Lequerica Cabello. **Clínica Universidad de Navarra. Pamplona.** Carlos Sánchez. **Hospital Universitario de Canarias. Tenerife.** Alberto Bravo, Lucrecia Rodríguez, Juana Escudero, Alejandro Morales, Maria Cruz Correa, Naybet Pérez. **Hospital Medina del Campo.** Juan Carlos Martín, Pilar Concejo, Juan Ramón Gómez, Clara Martínez, Javier Atienza. **Hospital Universitario Son Espases. Palma de Mallorca.** Alejandro Gil, Margarita Gamundi, Aina Ochogavía. **Hospital Universitario Son Llatzer.** **Palma de Mallorca.** Enrique Colás y Naila Pagés. **Hospital del Mar. Barcelona.** Susana González, Laia Cabré, Marta Pascual, Mayra Rebeka Abad. **Hospital Universitario Marqués de Valdecilla. Santander.** Isabel Seco, Víctor Valbuena, and Rubén Caina. **Hospital Universitario de Getafe.** Estefanía Sánchez, Alicia Ferrer, Virginia Jiménez. **Hospital Universitari Mútua Terrasa.** Salvadora Delgado and Mireia Lázaro. **Hospital Universitario de Cabueñes. Gijón.** Rubén Rodríguez, Adoración Meana y Paola Lora.
