# Supplementary figures and images for: The ILEOSTIM trial: A multicentre randomised controlled trial evaluating the impact of efferent loop stimulation prior to ileostomy reversal on postoperative ileus
Source: Colorectal Dis. 2026 May 6;28:e70448. doi: 10.1111/codi.70448 (PMC13150047; doi:10.1111/codi.70448)

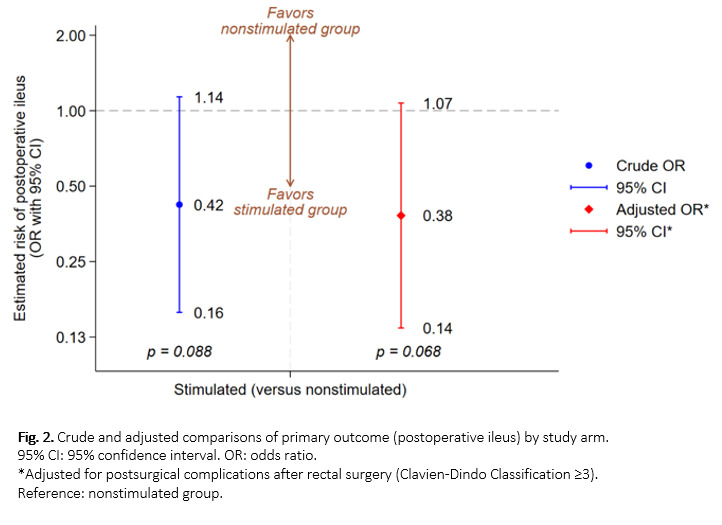

Supplement: Supplementary file 1 — Figure S1. Ileostomy stimulation. Figure S2. Crude and adjusted comparisons of primary outcome (postoperative ileus) by study arm. 95% CI, 95% confidence interval; OR, odds ratio. *Adjusted for postsurgical complications after rectal surgery (Clavien‐Dindo Classification ≥3). Reference: nonstimulated group. Data S1. CONSORT checklist. [file CODI-28-0-s001.zip › codi70448-sup-0002-FigureS2.jpg]

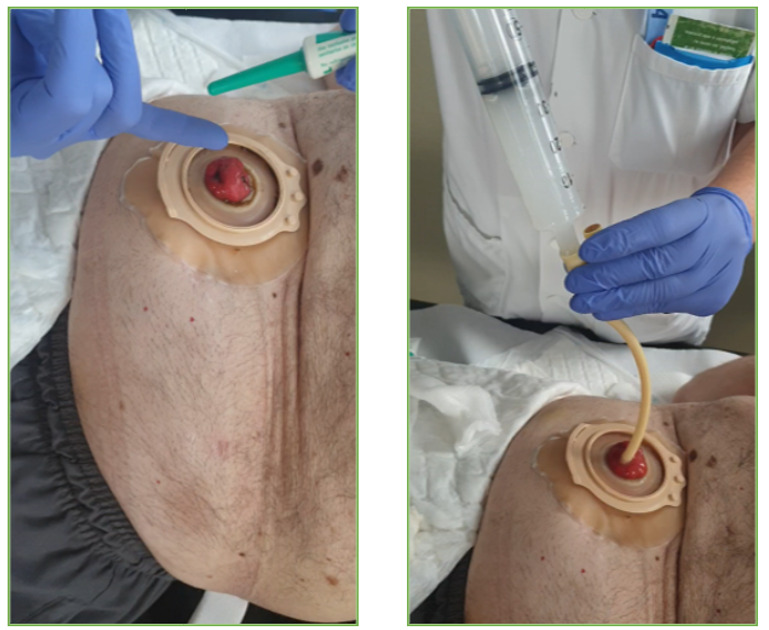

Supplement: Supplementary file 1 — Figure S1. Ileostomy stimulation. Figure S2. Crude and adjusted comparisons of primary outcome (postoperative ileus) by study arm. 95% CI, 95% confidence interval; OR, odds ratio. *Adjusted for postsurgical complications after rectal surgery (Clavien‐Dindo Classification ≥3). Reference: nonstimulated group. Data S1. CONSORT checklist. [file CODI-28-0-s001.zip › codi70448-sup-0001-FigureS1.jpg]
